# Genetic commonalities in eye and pigment evolution in the *Asellus aquaticus* species complex extend to the biogeographically and ecologically most divergent cave population, *A. infernus*

**DOI:** 10.1038/s41437-026-00868-z

**Published:** 2026-07-22

**Authors:** Serban M. Sarbu, Amélie Cayton, Gisselle Mejia, Navreet Raj, Natalie E. Oyler, Courtney Santos, Sofia Ugalde, LaNicia Ellis, Rupprit Kaur, Reanna Anderson, Noah Gibson, Kasdin Hamel, Micayla Flashner, Raluca Băncilă, Žiga Fišer, Meredith Protas

**Affiliations:** 1 Romanian Academy Cluj-Napoca Branch, “Emil Racoviţă” Institute of Speleology, Cluj-Napoca, Romania; 2 Department of Biological Sciences, California State University, Chico, CA USA; 3 “Emil Racoviţă” Institute of Speleology, Biospeleology and Karst Edaphobiology Compartment, Bucharest, Romania; 4 Department of Natural Sciences and Mathematics, Dominican University of California, San Rafael, CA USA; 5 Department of Biology, University of San Francisco, San Francisco, CA USA; 6 Biotechnical Faculty, Department of Biology, University of Ljubljana, Ljubljana, Slovenia

**Keywords:** Evolutionary genetics, Evolutionary biology

## Abstract

The *Asellus aquaticus* species complex, a freshwater isopod crustacean, includes surface and cave ecomorphs that differ markedly in eye and pigmentation phenotypes. Previous genetic studies in Slovenian cave populations revealed that the same genomic regions underlie eye and pigment loss across multiple cave populations, suggesting strong genetic constraints on these traits. To test the generality of this pattern, we examined *Asellus infernus*, a cave population from Mangalia, Romania, that inhabits thermal, sulfidic waters and is geographically distant from the Slovenian cave populations. Based on its distinct ecology and likely different ancestral surface population, we hypothesized that *A. infernus* would rely on different genomic regions for similar traits. Using hybrid crosses, we uncovered novel hybrid phenotypes unique to *A. infernus*, highlighting the presence of additional genetic variation not observed in Slovenian cave populations. However, we found that the same genomic regions previously implicated in Slovenian populations were also associated with similar eye and pigmentation phenotypes in *A. infernus*. Eye and pigment phenotypes examined were pigmented vs. unpigmented individuals, orange vs. red/brown and red vs. orange/brown eye pigment, stellate vs. diffuse head pigmentation pattern, and presence vs. absence of ommatidia and/or their fragments. These results point to shared genetic mechanisms of eye and pigment reduction between different cave populations. Whether these parallels reflect shared standing genetic variation in ancestral surface populations or convergent evolution through recurrent changes at the same loci due to pleiotropic fitness benefits remains unresolved.

## Introduction

The repeated evolution of similar traits has been documented across many biological systems such as stickleback fish and oldfield mice (Arendt and Reznick, 2008). Historically, uncovering the genetic mechanisms underlying repeated evolution has been challenging, as many species displaying these patterns are non-model organisms or emerging models with limited genomic resources. However, advances in sequencing technologies and molecular methods now allow genetic investigations beyond the traditional systems (Goldstein and Srivastava, 2022). Comparative studies reveal different possible outcomes: in some cases, the same genetic mechanisms are repeatedly recruited, such as the involvement of *mc1r* in pigmentation differences. In some cases, different mechanisms give rise to similar traits, such as in Bahamian mosquitofish (Bolnick et al., 2018). Distinct mutations in different genes may be more likely when genes are not constrained by pleiotropy (Gompel and Prud’homme, 2009), while different mutations within the same gene may occur at mutational “hotspots” (Courtier-Orgogozo, 2023). Conversely, identical mutations in the same gene across populations may indicate that standing genetic variation in the ancestral population drives repeated evolution (Schluter and Conte, 2009).

Cave animals provide excellent systems to study repeated evolution, as many species have independently evolved convergent traits such as eye reduction and pigment loss (Protas and Jeffery, 2012; Culver and Pipan, 2019). Because cave adaptation occurs in multiple, independently evolved lineages across the tree of life, one can examine repeated evolution across different scales—between distantly related species (e.g., shrimp and fish), between closely related species (e.g., two terrestrial isopods), or between populations within a single species or species complex. The best-studied cave animal in this context is the fish Mexican tetra, *Astyanax mexicanus* (Gross et al., 2023). Work across multiple populations has shown that mutations in the same gene underlie albinism in independently evolved populations (Protas et al., 2006; Gross et al., 2013; Klaassen et al., 2018), while additional pigmentation phenotypes are controlled by other loci (Gross et al., 2009, 2016; Riddle et al., 2020; Stahl et al., 2015). Eye loss may involve both similar and different mechanisms across populations (Borowsky 2008; Richards et al., 2026).

An emerging system for investigating repeated evolution of cave-associated traits is the *Asellus aquaticus* species complex, a freshwater isopod with both cave and surface ecomorphs distributed across Europe (Lafuente et al., 2021; Protas et al., 2023). Throughout this paper, we follow the population designations established by Protas et al. (2023) when referring to distinct cave populations. Previous studies in Slovenian populations have identified large effect genomic regions associated with eye and pigment loss (Protas et al., 2011; Re et al., 2018; Fišer et al., 2024). These populations span a range of geographic and phylogenetic distances, from adjacent parts within the same cave system (Old Subterranean Pivka and Subterranean Rak populations) to more distant and distinct cave systems (*Asellus kosswigi* and Subterranean Krka populations). Remarkably, across all populations examined to date, the same genomic regions have been implicated in eye and pigment traits (Protas et al., 2011; Re et al., 2018; Rodas et al., 2023; Fišer et al., 2024). This striking genetic parallelism is evident even when the trait itself is not expressed in cave animals. For example, orange pigmentation segregates in crosses of distinct cave populations with surface animals, despite not being visible in the cave isopods themselves because it is masked by loss of pigmentation (Protas et al., 2011). The causes of this remarkable genetic constraint remain unresolved.

Several factors could influence whether the same or different mechanisms underlie repeated evolution. Cave populations derived from the same founding surface population shared its standing genetic variation and might have recruited the same alleles during adaptation, whereas those derived from different founding lineages and gene pools may be more likely to fix alternative alleles of the same or different genes (Bohutínská and Peichel, 2024). Similarly, environmental context may shape evolutionary outcomes (Schluter, 2000). Although all subterranean habitats, including caves, are characterized by the absence of light, it is increasingly recognized that they may differ in many other environmental factors, such as temperature, humidity, food quality and quantity, and overall environmental stability (Pipan and Culver, 2012; Fišer et al., 2023; Robertson et al., 2023). Populations adapting to ecologically similar caves may rely on the same genetic mechanisms, while populations in ecologically divergent caves might require different genetic solutions, especially if pleiotropic or linked alleles must simultaneously respond to multiple selective pressures. To date, all studied cave populations in Slovenia of the *A. aquaticus* species complex are located within ca. 100 km of each other (Verovnik et al., 2009; Protas et al., 2023). Moreover, the Slovenian caves examined so far share broadly similar environments, underground stretches of sinking rivers, with non-sulfidic waters, seasonal temperature fluctuations between ca. 7 °C and 13 °C, and comparable food resources (Sket, 1994; Protas et al., 2023).

Here, we extend this framework to *Asellus infernus*, arguably the most divergent member of the *A. aquaticus* species complex (Turk-Prevorčnik and Blejec, 1998; Konec et al., 2015). *A. infernus* inhabits a groundwater ecosystem near Mangalia in Romania, over 1000 km away from the Slovenian populations. This geographic separation is mirrored in phylogeny: whereas the Slovenian populations belong to the Northern Dinaric Clade, *A. infernus* is part of the Central European Clade (Konec et al., 2015; Protas et al., 2023), and thus clearly derives from a different founding surface lineage. Beyond its phylogenetic distinctness, *A. infernus* is set apart by its striking ecological conditions. Its subterranean habitat is characterized by highly constant sulfidic and thermal waters (ca. 21–23 °C) (Sarbu et al., 1996, 2018). These extremes also shape its biology: individuals feed primarily on sulfur-oxidizing microbial biofilms and face comparatively limited predation (e.g., from the waterscorpion *Nepa anophthalma* and the amphipods *Niphargus racovitzai* and *Niphargus dancaui*) (Brad et al., 2021). In contrast, Slovenian populations consume detritus and biofilms and experience stronger predation pressure (e.g., from the olm *Proteus anguinus*, multiple *Niphargus* species, and possibly leeches) (Fišer et al., 2019; Benko et al., 2024). Although *A. infernus* has historically been challenging to study, recent work has established transcriptomic resources, enabled comparative embryology, and produced F₁ crosses (Rodas et al., 2023; Lomheim et al., 2025). Complementation crosses further suggest that the same genomic region underlies both pigmentation loss and orange pigmentation in *A. infernus* and Slovenian populations (Rodas et al., 2023). However, testing additional genotype–phenotype associations has so far not been possible without generating F₂ hybrids.

In this study, we test whether the remarkable genetic parallelism observed in Slovenian cave populations of the *A. aquaticus* species complex extends to *A. infernus*, a biogeographically and ecologically distinct cave population. Specifically, we generate F₂ individuals from *A. infernus* crossed to the surface ecomorph, describe their eye and pigment phenotypes, and assess whether these are associated with the same genomic regions as in Slovenian cave populations. This approach allows us to ask whether the genetic bias toward repeated use of the same genomic regions persists under substantially different evolutionary contexts, or whether novel mechanisms emerge.

## Materials and methods

### Animal sampling and husbandry

For this study, we collected a cave and a surface ecomorph of the *Asellus aquaticus* species complex from the vicinity of Mangalia, Romania. Around 30 individuals of each were collected. The cave ecomorph, *Asellus infernus*, was sampled in May 2021 from Avram Iancu Well and Dimitru Ana Well, both characterized by sulfidic, thermal water (ca. 21–23 °C). The surface ecomorph, *A. aquaticus*, was collected from the Turkish Bath stream in July 2018, and from Limanu Bridge Stream in May 2021. These latter sites are located along the shore of the same pond and were treated as a single panmictic population; both contain non-sulfidic water.

Animals were maintained following the protocol of Protas et al. (2011), fed algae pellets, and reared in artificial *Daphnia* water (Lynch et al., 1986). Both ecomorphs were kept under identical laboratory conditions as described by Lomheim et al. (2025).

### Genetic crosses

**Fig. 1:**
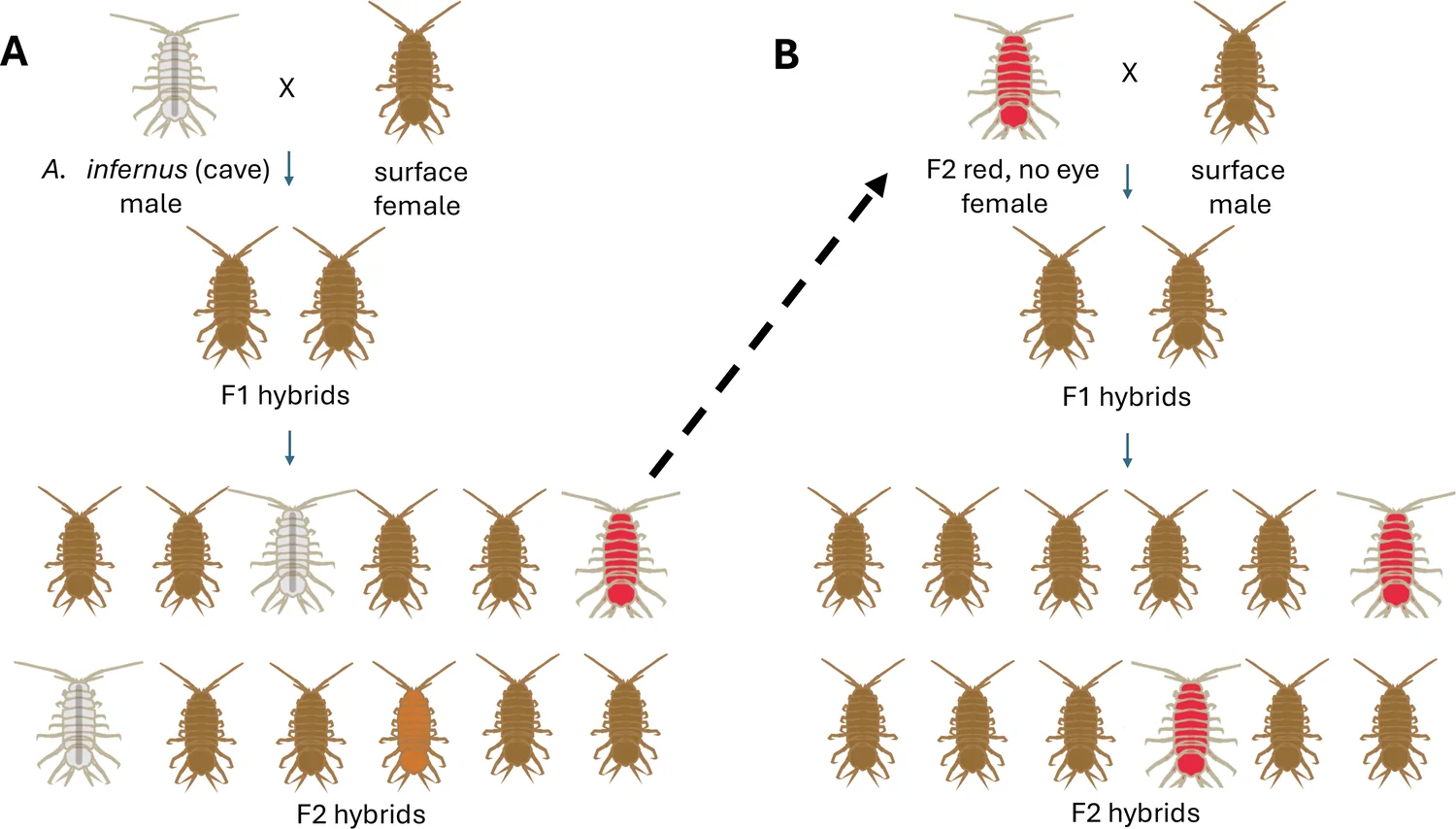
Genetic crosses used in this study. **A** *Classical cave* *×* *surface F₂ cross*: cave males of *Asellus infernus* were crossed with surface females to produce F₁ hybrids, which were intercrossed to generate F₂ hybrids. These F₂ individuals exhibited high variation in eye and head pigmentation and eye (ommatidia) phenotypes. **B** *Red-eyeless × surface F₂ cross*: an F₂ female from the classical cross (red eye pigment, no ommatidia and/or ommatidial fragments) was crossed with a surface male. The resulting F₁ hybrids were intercrossed to produce F₂ offspring.

We first established a *classical cave* *×* *surface F₂ cross* (Fig. 1A). Cave males (*A. infernus*) were crossed with surface females. In total, three different surface females and three different cave males were used; each cross had a unique set of parents. Three initial F₁ crosses were produced, which in turn yielded eight, nine, and ten F₂ families, respectively. In two of these initial F₁ crosses, the surface female parent was likely heterozygous for the orange eye pigmentation phenotype. These F₁ animals were originally generated to test whether the orange pigmentation observed in different populations was controlled by the same or distinct genomic regions (see Rodas et al., 2023). In total, 120 F₂ individuals were obtained, ranging from 4 to 8 months of age when harvested for phenotyping and genotyping.

Phenotypic variation was pronounced in the *classical cave* *×* *surface F₂ cross*, making certain traits—such as orange versus red eye pigmentation—difficult to distinguish. Generally, red and orange are distinct phenotypes, but in the background of additional pigmentation differences, some individuals seemed intermediate between the two phenotypes. One possible reason for this is that multiple eye and pigmentation traits could exhibit epistatic interactions (unequal interactions between different genetic loci). Notably, pigmentation phenotypes were more diverse than those previously observed in Slovenian populations. To reduce potential confounding from epistasis and clarify phenotype–genotype associations, we generated additional crosses using F₂ individuals of specific phenotypes backcrossed to surface individuals. Among these, the *red-eyeless* *×* *surface F₂ cross* (Fig. 1B) was an F₂ female with red eye pigmentation and no ommatidia from the *classical cave* *×* *surface F₂ cross* crossed with a surface male. Thirteen F₁ families were established, which subsequently yielded 57 F₂ individuals. These offspring were harvested at 4–12 months of age for phenotyping and genotyping. This cross was selected because it produced the largest number of offspring and involved two phenotypes—red eye pigmentation and stellate pigmentation—that did not show statistically significant associations in the classical F₂ population.

### Phenotyping

Animals were first anesthetized in a clove oil solution (50 mL artificial *Daphnia* water with 20 µL clove oil) and rinsed twice in artificial *Daphnia* water, following Mojaddidi et al. (2018). Each animal was then photographed under a Leica S8 Apo microscope. For phenotyping, heads were carefully removed with forceps from the rest of the body. Heads were scored for the following qualitative traits: (i) eye pigment (brown, orange, red, no pigment), (ii) head pigment (present or absent), (iii) head pigmentation pattern (stellate or diffuse), (iv) eye structure (number of ommatidia or ommatidial fragments on both sides of the head). The cave parental phenotype is no eye pigment, no head pigment, and no ommatidia or ommatidial fragments. The surface parental phenotype is brown eye pigment, brown diffuse head pigment, and four ommatidia in each eye. Using these phenotypic data we later tested the following genotype–phenotype associations: 1) presence versus absence of pigment (present indicated any pigment in the eyes or head, while absent indicated no pigment in either), 2) orange versus red/brown eye pigment, 3) red versus orange/brown eye pigment, 4) stellate versus diffuse head pigmentation pattern, 5) presence versus absence of ommatidia and/or ommatidial fragments. Individuals were classified as “eyeless” if they had no ommatidia or ≤2 ommatidial fragments (often unilateral, occasionally with a small bump counted as a fragment). Individuals with ommatidia or >2 ommatidial fragments were classified as having ommatidia and/or fragments. Ambiguous phenotypes were not included in the phenotype/genotype associations. After phenotyping, heads and telsons were preserved in 100% ethanol, while the rest of the body was retained for DNA extraction.

### Genotyping

We targeted genetic markers in the following genes: *scarlet* (presence versus absence of eye and/or head pigmentation), *pax2* (red versus orange/brown eye pigmentation), *nckx30* (orange versus red/brown eye pigmentation), and both *sob* and *fat facets* (presence versus absence of ommatidia and/or their fragments, and stellate versus diffuse pigmentation pattern) (Protas et al., 2011; Re et al., 2018; Fišer et al., 2024). These markers were selected as they mark the regions responsible for each of the corresponding phenotypes, though we do not currently know the actual genes responsible for the phenotypes.

DNA was extracted from the body or legs of each individual using the QIAamp Mini or Micro Kit (Qiagen). PCR was performed with primers listed in Table 1. For *sob* and *fat facets*, the same SNPs were used across both the *classical cave* *×* *surface F₂ cross* and the *red-eyeless* *×* *surface F₂ cross*. In contrast, the SNP used for *pax2* in the classical cross was not polymorphic in the red-eyeless cross. Instead, a different polymorphism with four alleles (two unique to the red-eyeless individual and two unique to the surface individual) was employed. PCR products were purified with ExoSAP-IT and sequenced at MCLAB. Genotypes were determined by chromatogram inspection using FinchTV v1.4.0 (Geospiza, Inc.).

**Table 1:** PCR primers used to amplify genetic markers in *scarlet*, *sob*, *fat facets*, *nckx30*, and *pax2* in *Asellus* F_2_ individuals.

Genetic marker	
*scarlet* (=aa17 in Protas et al. (2011))	
Forward primer	TAGCGCGACATGTTCTGG
Reverse primer	CTAGATTAGGCATTGCTCAT
Polymorphism used for genotyping	ACAGAACTC**[C/-]**ACATT
*sob*	
Forward primer	CTCCTCCATGTGCAGGATCTTATGG
Reverse primer	CCGTATGTATTTCAGTTCAATATGTCG
Polymorphism used for genotyping	TTCCACACA**[T/C]**TTGAACGGCTT
*fat facets* (=aa86 in Protas et al. (2011))	
Forward primer	TGCTGGCAAGTTTTCTCTATGGCACT
Reverse primer	CAGAATGCAAAATGGATGATGATGA
Polymorphism used for genotyping	ACATTGGTT**[C/T]**TTTATTTC
*nckx30* (=aa50 in Protas et al. (2011))	
Forward primer	TCCTCCGAGATCTGCAACTTCTCTA
Reverse primer	GTCTTCGCTTGTCCAAATGACGATA
Polymorphism used for genotyping	CCTTGGGCG**[C/T]**CGTCGC
*pax2* (=aa81 in Protas et al. (2011))	
Forward primer	TACAAGAGAGAAAACCCGACGATGT
Reverse primer	GGATAGGAGGAAGGAGTGCTGGTTT
Polymorphism used for genotyping	*classical cave* *×* *surface F*_*2*_ *cross*: CCTTGGAGCACAC**[C/T]**CTCCTGCCTGCTTTTGTT *red-eyeless* *×* *surface F*_*2*_ *cross*: GCACTT**[T/A]**TATTT [InfA/SurfA] : GCACTTA--TTTC **[G/A]**TG [InfB/SurfB]

Polymorphism used for genotyping is marked in bold, and several nucleotides before and after it are shown. In brackets, nucleotides before the slash indicate the cave allele, while nucleotides after the slash indicate the surface allele. A “-“ indicates that a nucleotide was missing and that there was a size polymorphism between the alleles. *Scarlet* and *nckx30* were only analyzed for the *classical cave × surface F*_*2*_ *cross* as the *red-eyeless* *×* *surface F*_*2*_ *cross* resulted in too few unpigmented or orange individuals for further analysis. *Sob*, *fat facets*, and *pax2* were genotyped in both cross types. For *sob* and *fat facets*, the same polymorphism was used in both cross types. In *pax2*, the parents were fixed for the polymorphism used in the *classical cave* *×* *surface F*_*2*_ *cross*. Therefore, another polymorphism was genotyped using the same primer pairs. Interestingly, there were a total of four different alleles present in the two parents; each parent was heterozygous but with two different alleles than the other parent. Both parents had a long and a short allele that differed by two base pairs. In addition to the difference in size, there were also unique SNPs present in each sequence such that the surface parent had two different alleles, and the red-eyeless F_2_ individual had two different alleles (see Figure S1 for details). The two alleles of the red-eyeless F_2_ parent were called InfA and InfB, and the two alleles of the surface parent were called SurfA and SurfB.

### Statistical Analysis

Fisher’s exact tests were used to assess associations between each genetic marker and the corresponding phenotypic traits. The strength of associations was quantified using Cramér’s V (0 = no association, 1 = perfect association). Statistical power (1-β) for each Fisher’s exact test was estimated using Monte Carlo simulation (100,000 iterations). Contingency tables were repeatedly generated from the observed cell probabilities using multinomial sampling, and the proportion of simulations with *p* < 0.05 was taken as the estimated power. All analyses were conducted in R v4.0.2 (R Development Core Team, 2022).

## Results

**Fig. 2:**
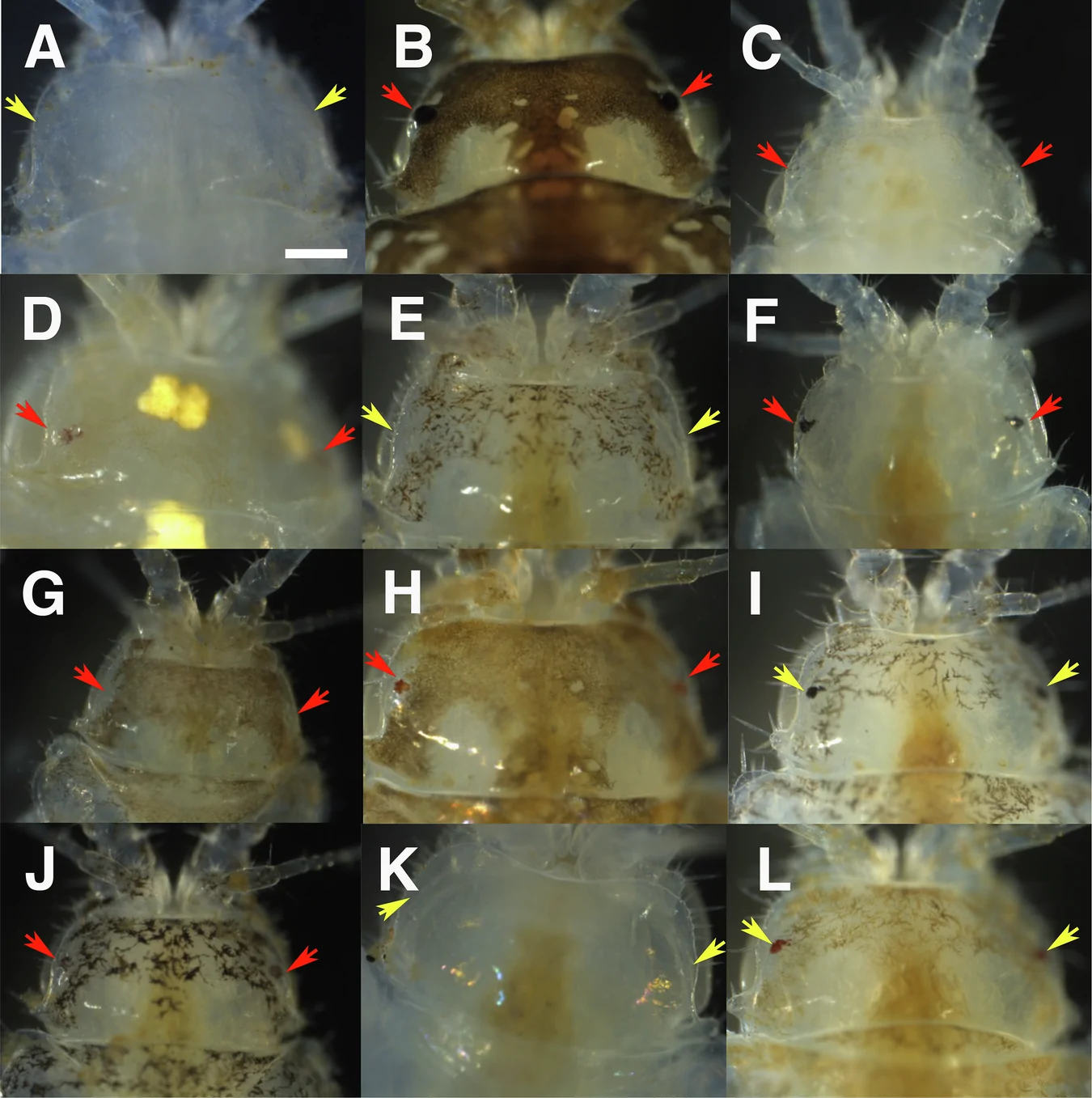
Pigmentation phenotypes in F₂ individuals from the *classical cave* *×* *surface F₂ cross.* *A. infernus* individual showing no ommatidia and no pigmentation (**A**) from Lomheim et al., 2025. Surface individual showing brown, diffuse pigmentation and ommatidia (**B**). F₂ hybrids exhibited diverse phenotypes, including diffuse pigmentation pattern (**D**, **G**, **H**), stellate pigmentation pattern (**E**, **I**, **J**, **L**), complete absence of eye and head pigmentation (**K**), head pigmentation without eye pigment (**C**, **E**), and minimal or no head pigmentation with eye pigment (**F**). Eye pigment color also varied, appearing as orange/red (**D**, **H**, **L**) or brown (**F**, **I**, **J**). Scale bar in A is 0.2 mm and applies to all panels. Yellow arrows signify no ommatidia (though pigmented eye spots might be present), and red arrows signify ommatidia.

**Fig. 3:**
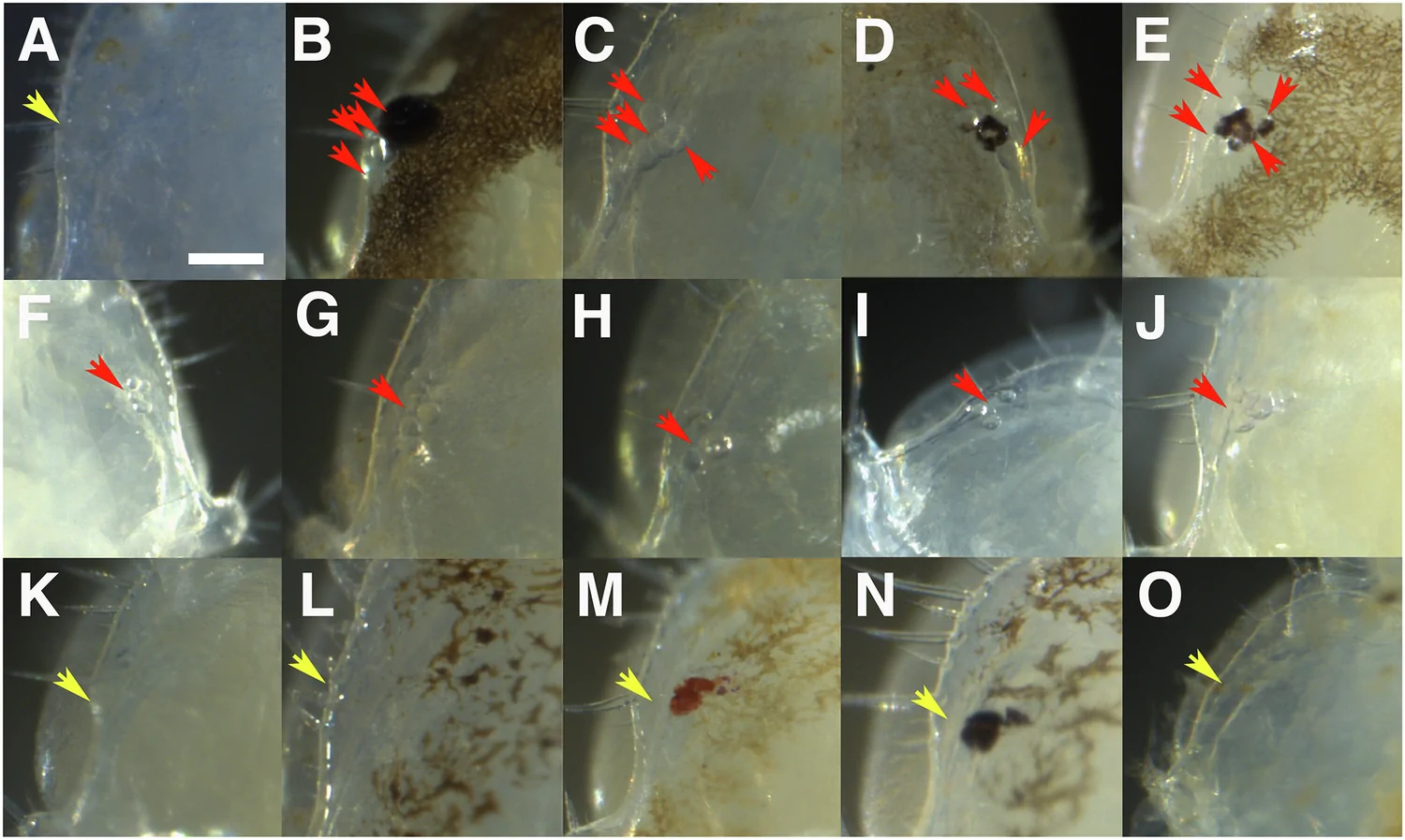
Eye (ommatidia) phenotypes in F₂ individuals from the *classical cave* *×* *surface F*_*2*_*cross.* *A. infernus* individual showing no ommatidia and no pigmented eye spot (**A**) from Lomheim et al., 2025. Surface individual showing 4 ommatidia and a brown pigmented eye spot (**B**). Observed phenotypes in F_2_ included: three-four normal ommatidia (sometimes the fourth ommatidia is difficult to see if obscured by pigmentation) (**C**–**E**), fragmented ommatidia or a mixture of normal and fragmented ommatidia (**F**–**J**), and complete absence of ommatidia or presence of only 1–2 ommatidial fragments (**K**–**O**). Scale bar in A is 0.1 mm and applies to all panels. Yellow arrows signify no ommatidia (though pigmented eye spots might be present) and red arrows signify either a single ommatidia or fragmented/misshapen ommatidia.

The *classical cave* *×* *surface F₂ cross*, generated by mating cave males of *Asellus infernus* with surface females of *A. aquaticus* (Fig. 1A), produced F₂ hybrids with highly variable pigmentation and eye (ommatidia) phenotypes (Figs. 2, 3). When pigmentation was present, it differed in color in the eye (brown, orange, or red), pattern (stellate or diffuse), and body parts that were pigmented (eye and/or head) (Fig. 2). Eye phenotypes ranged from normal ommatidia to fragmented ommatidia, a mixture of both, or a complete absence of ommatidia or their fragments (Fig. 3). Most of these phenotypes had previously been observed in crosses with Slovenian cave populations (Protas et al., 2011; Re et al., 2018; Fišer et al., 2024) (Fig. 2D, H, I, J, K, L). However, novel phenotypes were also detected, including head pigmentation without eye pigment (Fig. 2E, G), and minimal head pigmentation (Fig. 2C, F).

The phenotypically diverse F₂ individuals were then used to test seven genotype–phenotype associations (Table 2), focusing on the same or similar traits examined in earlier studies of Slovenian cave populations (Protas et al., 2011; Re et al., 2018; Fišer et al., 2024). *Scarlet* genotype showed a statistically significant association with presence versus absence of pigment, while *nckx30* was statistically significantly associated with orange versus red/brown eye pigment. For *pax2*, the association with red versus orange/brown eye pigment was marginally statistically significant (*p* = 0.056). Both *sob* and *fat facets* genotypes were statistically significantly associated with presence versus absence of ommatidia and/or ommatidial fragments. Interestingly, at *sob* the genotypic distribution deviated from expectations, with most eyeless individuals being heterozygous rather than homozygous for the cave allele. Finally, *sob* also showed a marginally statistically significant association with stellate versus diffuse head pigmentation (*p* = 0.065), whereas *fat facets* showed no statistically significant association with this trait. All association tests with marginal or no statistical significance had low statistical power (<0.60).

**Table 2:** Genotype–phenotype associations in F_2_ individuals of the *classical cave* *×* *surface F*_*2*_ *cross*.

Association				Fisher’s exact test	Cramér’s V	Power (1 - β)
*scarlet*	**CC**	**CS**	**SS**			
unpigmented	23	3	0	*p* < 0.001	0.804	> 0.999
pigmented^a^	5	56	19			
*nckx30*						
orange eye pigment	4	6	1	*p* = 0.039	0.367	0.631
red/brown eye pigment	3	28	12			
*pax2*						
red eye pigment	2	3	2	*p* = 0.056	0.334	0.561
orange/brown eye pigment	2	35	32			
*sob*						
no ommatidia and/or fragments^b^	4	10	3	*p* < 0.001	0.594	0.999
with ommatidia and/or fragments	20	4	54			
*sob*						
stellate	3	4	3	*p* = 0.065	0.274	0.542
diffuse	11	8	34			
*fat facets*						
no ommatidia and/or fragments^b^	6	8	0	*p* < 0.001	0.41	0.979
with ommatidia and/or fragments	6	56	23			
*fat facets*						
stellate	3	7	0	*p* = 0.21	0.225	0.241
diffuse	9	34	13			

F_2_ hybrids were either homozygous for the cave allele (CC), heterozygous (CS), or homozygous for the surface allele (SS). Cramér’s V estimates the strength of association between the genotype and phenotypes measured. Statistical power (1-β) was estimated as the proportion of Monte Carlo simulations (100,000 iterations) with *p* < 0.05.

^a^pigment present either in head, eye, or both.

^b^includes also individuals with 1–2 ommatidial fragments (see Methods).

**Fig. 4:**
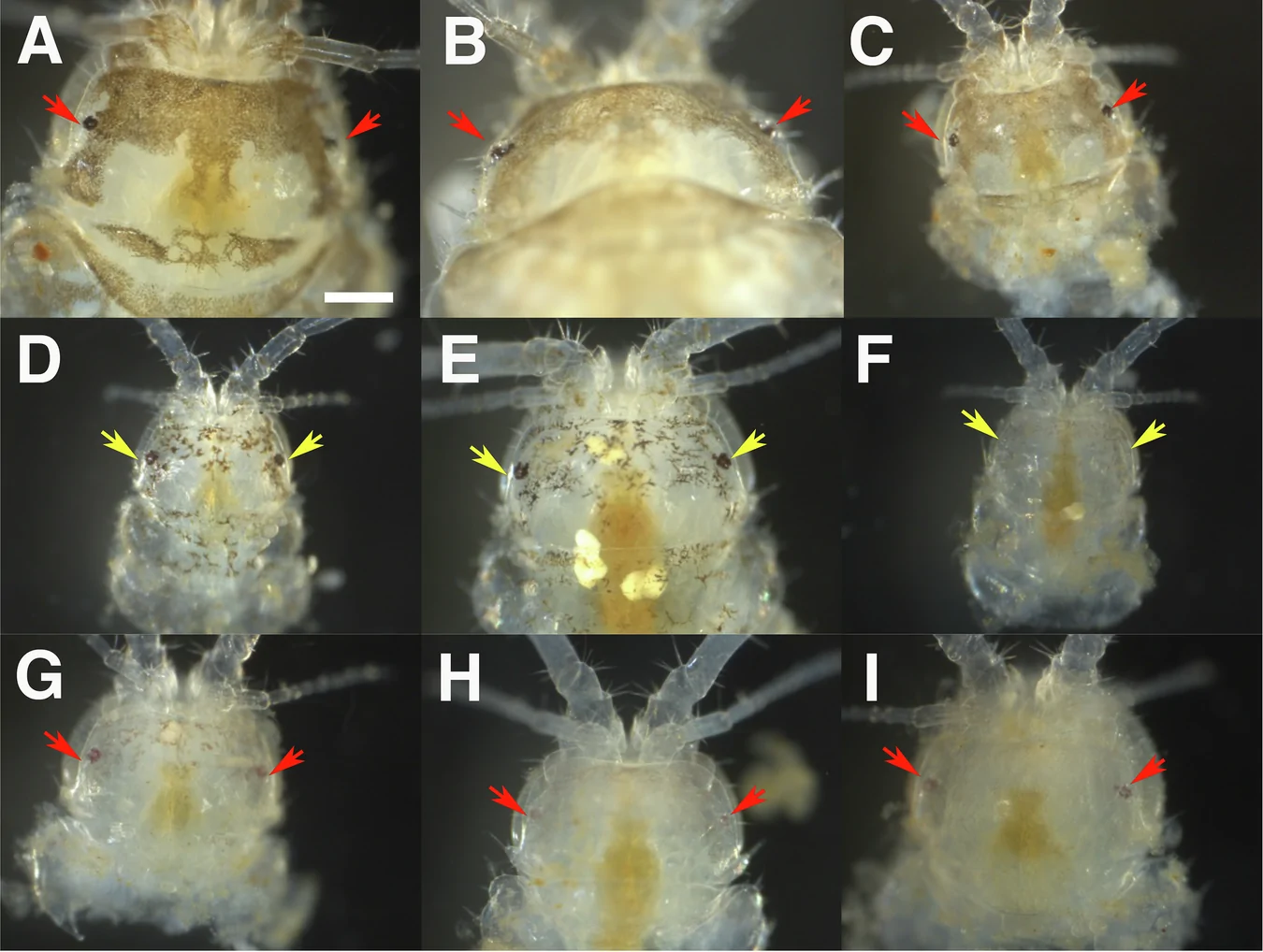
Pigmentation phenotypes in F₂ individuals from the *red-eyeless* *×* *surface F₂ cross.* F₂ hybrids exhibited either brown (**A**–**E**) or red (**F**–**I**) eye pigment, with head pigmentation pattern that was either diffuse (**A**, **B**, **C**, **H**, **I**) or stellate (**D**, **E**, **G**). Scale bar in A is 0.2 mm and applies for all panels. Yellow arrows signify no ommatidia (though pigmented eye spots might be present) and red arrows signify ommatidia.

**Fig. 5:**
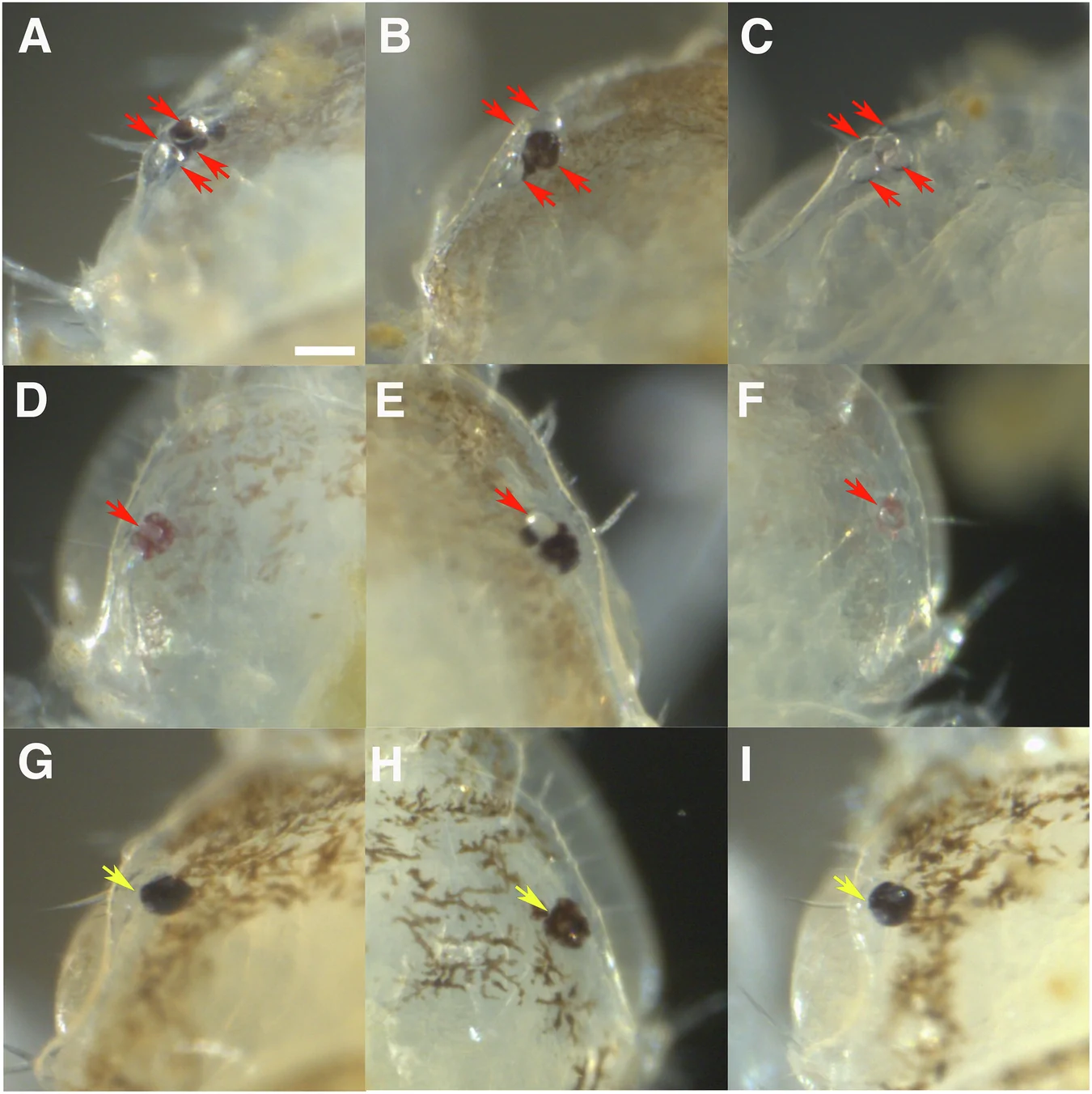
Eye (ommatidia) phenotypes in F_2_ individuals of the red-eyeless × surface F_2_ cross. Observed phenotypes included: normal ommatidia (**A**, **B,** **C**), fragmented ommatidia or a mixture of normal and fragmented ommatidia (**D**, **E**, **F**), and a complete absence of ommatidia or presence of only 1–2 ommatidial fragments (**G**, **H**, **I**). Scale bar in A is 0.1 mm and applies to all panels. Yellow arrows signify no ommatidia (though pigmented eye spots might be present) and red arrows signify either a single ommatidia or fragmented/misshapen ommatidia.

In the *red-eyeless* *×* *surface F₂ cross* (Fig. 1B), an F₂ female with red eye pigmentation and no ommatidia (from the classical cross) was crossed with a surface male. The resulting F₂ offspring exhibited three main phenotypic contrasts: red versus brown eye pigment, stellate versus diffuse head pigmentation, and presence versus absence of ommatidia and/or ommatidial fragments (Figs. 4, 5). Five genotype–phenotype associations were tested (Table 3). The *pax2* genotype was statistically significantly associated with red versus brown eye pigment. *Sob* was statistically significantly associated with both ommatidia presence/absence and stellate versus diffuse head pigmentation pattern. *Fat facets* showed a marginally statistically significant association with ommatidia presence/absence (*p* = 0.065) and no statistically significant association with stellate versus diffuse head pigmentation pattern. These last two association tests also had low statistical power (<0.50).

**Table 3:** Genotype–phenotype association in F_2_ individuals of the *red-eyeless* *×* *surface F*_*2*_ *cross*.

Association				Fisher’s exact test	Cramér’s V	Power (1 - β)
*pax2*	CC	CS	SS			
red eye pigment	5	0	0	*p* < 0.001	0.902	> 0.999
orange/brown eye pigment	1	31	10			
*sob*						
no ommatidia and/or fragments^a^	4	6	1	*p* < 0.001	0.579	0.954
with ommatidia and/or fragments	0	21	15			
*sob*						
stellate	6	5	1	*p* < 0.001	0.669	0.993
diffuse	0	24	15			
*fat facets*						
no ommatidia and/or fragments ^a^	6	1	0	*p* = 0.065	0.4017	0.480
with ommatidia and/or fragments	10	7	9			
*fat facets*						
stellate	7	1	0	*p* = 0.11	0.377	0.404
diffuse	12	7	8			

F_2_ hybrids were either homozygous for the F_2_ female parent (red eye pigment and without normal or fragmented ommatidia; a product of the *classical cave* *×* *surface F*_*2*_ *cross*) allele, heterozygous (CS), or homozygous for the surface allele (SS). For *pax2*, both original parents were heterozygous (see Methods and Fig. S1) with a total of four different alleles, InfA and InfB for the F2 parent and SurfA and SurfB for the surface parent. Here, we have C represent either InfA or InfB and S represent either SurfA or SurfB. Cramér’s *V* estimates the strength of association between the genotype and phenotypes measured. Statistical power (1-β) was estimated as the proportion of Monte Carlo simulations (100,000 iterations) with *p* < 0.05.

^a^Includes also individuals with 1–2 ommatidial fragments (see Methods).

## Discussion

In this study, we crossed individuals from the Romanian cave population *Asellus infernus* with surface *A. aquaticus* to generate F₁ and subsequently F₂ hybrids. Genotype–phenotype association tests revealed that the same genomic regions previously linked to eye and pigment phenotypes in Slovenian cave populations (Protas et al., 2011; Re et al., 2018; Fišer et al., 2024) are also associated with the same traits in *A. infernus* (Table 4). This finding is striking given the considerable biogeographic and ecological distinctness of *A. infernus* compared to the Slovenian cave populations.

**Table 4:** Convergence of genomic regions associated with pigment and eye phenotypes in the *Asellus aquaticus* species complex. Updated from Fišer et al. (2024).

Cave population	Pigment: present versus absent	Eye pigment: orange versus red/brown	Eye pigment: red versus orange/brown	Pigmentation pattern: stellate versus diffuse	Eye (ommatidia): present versus absent
Old Subterranean Pivka	Y	Y	Y	Y	Y
Subterranean Rak	Y	Y	Y	Y	Y
*Asellus infernus*	Y	Y	Y	Y	Y
*Asellus kosswigi*	Y	Y	?	?	?
Subterranean Krka	Y	NA	NA	NA	NA

Data from Protas et al. (2011), Re et al. (2018), Rodas et al. (2023), Fišer et al. (2024), and this paper.

*Y* - The same genomic region is responsible for the phenotype in all populations marked, *?* - not tested or too small samples for reliable assessment, *NA* - phenotype not observed in F2 individuals and therefore not tested.

For the phenotypes of pigment absence and orange eye pigment, the results were consistent with those from the Slovenian cave populations, Old Subterranean Pivka and Subterranean Rak (Re et al., 2018). The pigmented versus unpigmented phenotype and the genomic region marked by *scarlet* showed a significant and similarly strong association (as measured by Cramér’s *V*) in *A. infernus*. Complementation crosses provide further support: a cross between a Subterranean Rak hybrid and *A. infernus*, as well as crosses among multiple Slovenian cave populations, indicate that the same gene likely underlies pigment loss across all populations examined (Protas et al., 2011; Re et al., 2018; Rodas et al., 2023; Fišer et al., 2024). The genomic region marked by *nckx30* was also significantly associated with the orange eye pigment phenotype in *A. infernus*. The lower Cramér’s *V* observed in *A. infernus* (0.367), compared to the results from Slovenian cave populations, was expected, as some surface females used for the F₂ crosses were heterozygous for an orange allele (see Methods; Rodas et al., 2023). Previous complementation tests further support that the same gene likely underlies the orange eye pigment phenotype across both cave and surface populations in Romania and Slovenia (Rodas et al., 2023).

For the red eye pigment as well as eye (ommatidia) and head pigmentation pattern phenotypes, associations with *pax2* and *sob*, respectively, were more variable and less consistent with those from the Slovenian cave populations. For the red eye pigment phenotype, the *classical cave* *×* *surface F₂ cross* showed a marginally significant and weak association with *pax2* (*p* = 0.056, *V* = 0.334), in contrast to the Old Subterranean Pivka and Subterranean Rak populations where the association was strong and significant (Re et al., 2018). By contrast, the *red-eyeless* *×* *surface F₂ cross* revealed a significant and strong association of the phenotype of red eye pigment versus orange/brown eye pigment with *pax2*. A similar pattern was observed for *sob*: while both crosses showed a significant association with eye loss, only the *red-eyeless* *×* *surface F₂ cross* revealed a significant and strong association with head pigmentation pattern. These differences are probably due to the low statistical power of these specific tests for the classical cross (<0.60) but also likely reflect the influence of multiple loci, potentially interacting epistatically, on pigmentation phenotypes in *A. infernus*. Restricting the analysis to a subset of variation in the *red-eyeless* *×* *surface F₂ cross* may have reduced this complexity, uncovering stronger associations. Thus, our results also demonstrate the value of targeted F₂ designs—these are especially useful when multiple loci contribute non-additively to a trait. More surprising was the association of the heterozygous *sob* genotype with the eyeless phenotype in the *classical cave* *×* *surface F₂ cross*. At least one of the cave parents was heterozygous rather than homozygous for the cave allele, which may partially explain this result. However, it remains unclear why heterozygotes were disproportionately associated with eye loss. Potential explanations include structural variation such as inversions or translocations between cave and surface genomes, or selective survival of recombinants carrying compatible genomic regions.

The associations of eye (ommatidia) and head pigmentation pattern with *fat facets* were the least concordant with those from the Slovenian cave populations. Notably, *fat facets* showed no significant association with head pigmentation pattern in either cross, likely due to low statistical power of these tests (<0.41). It was, however, significantly but weakly associated with eye loss in the classical cross, and showed marginal significance (*p* = 0.065) for the same association in the red-eyeless cross. This latter discrepancy may again reflect a statistical artefact: the smaller sample size for *fat facets* in the red-eyeless cross translated to reduced statistical power (0.979 versus 0.480). Alternatively, we see two biological explanations. First, the markers tested are proxies and not the causal loci themselves, so recombination in the red-eyeless cross could have disrupted linkage. Second, inspection of the genotype distributions in the red-eyeless cross revealed a strong bias toward homozygous cave and heterozygous genotypes, with very few surface homozygotes. Genotyping of F₁ individuals further confirmed an unexpected pattern: some F₁s were homozygous for the cave allele despite having one surface and one cave parent, both homozygous for their respective alleles. A possible explanation is that this genomic region is haploid in some individuals, e.g., located on a sex chromosome. Existing evidence suggests that in *Asellus*, males are likely the heterogametic sex (Rocchi et al., 1984; Volpi et al., 1992), although the current knowledge is insufficient and things are complicated due to the common occurrence of sex changes via *Wolbachia* infestation (Vitagliano et al., 1994; Bouchon et al., 1998). Because our current genotyping cannot distinguish between hemizygous and homozygous states, such cases could produce misleading results. Resolving this will require methods capable of discriminating among all possible allele states, as well as sexing the F₁ hybrids.

Overall, the genomic regions previously identified in Slovenian populations appear to also play a role in *A. infernus*. However, it is likely that additional regions might also be involved in these traits; a genome-wide mapping approach will be necessary to uncover these. In the future, when genome-wide markers are identified between the Romanian cave and surface populations, it will be possible to perform QTL mapping of these traits. Additionally, the Romanian cave population reveals additional complexity not observed in the Slovenian cave hybrids. Unique pigmentation combinations were observed in *A. infernus* F_2_ hybrids, such as individuals with body and head pigmentation but no eye pigment, or individuals with dark eye pigment but little body or head pigmentation—phenotypes absent in Slovenian hybrids. Similar to the Slovenian populations, however, *A. infernus* hybrids displayed a wide spectrum of eye structure phenotypes, including individuals with intact ommatidia, ommatidial fragments, or various combinations of the two (Protas et al., 2011; Re et al., 2018). These differences could arise because not all relevant variation is fixed within cave populations, so the outcome of crosses depends on which individuals are sampled. Alternatively, *A. infernus* may harbor additional genetic variation not found in Slovenian cave populations. Identifying these novel loci will require genome-wide mapping approaches, but also a more robust breeding program. Historically, *A. infernus* has been difficult to maintain in the laboratory, and previous attempts with independent collections produced few or no F₂ offspring. In contrast, the batch described here produced robust F₁s and two lab-bred generations of cave × cave crosses. The greater number of F₂s may reflect the larger F₁ pool, but cave individuals in this collection also appeared more robust than in past efforts. Why this batch was more successful remains unclear. Future studies could test whether reproductive success varies with collection timing or with conditions better mimicking the natural sulfidic, thermal environment.

The observed genetic constraint for the same genomic regions underpinning eye and pigment phenotypes in both Slovenian cave populations and *A. infernus* is particularly striking given the highly distinctive nature of the latter. *A. infernus* is geographically distant (>1000 km), belongs to a different phylogenetic clade, and inhabits thermal (21 –23 °C), sulfidic waters (Konec et al., 2015; Sarbu et al., 2018; Protas et al., 2023). It primarily feeds on sulfur-oxidizing microbial biofilms and experiences minimal predation (Brad et al., 2021), in contrast to Slovenian cave populations, which mainly consume allochthonous detritus and face comparatively stronger predation from the olm, *Proteus anguinus* (Fišer et al., 2019; Benko et al., 2024). One explanation for genetic parallelism is that some of the causative variation derives from common standing genetic variation in surface populations (Rodas et al., 2023). Although Slovenian and Romanian surface lineages belong to different clades of the *A. aquaticus* species complex, the population size of both is large, and they dwell in similar environmental conditions. Thus, with minimal genetic drift and divergent selection, they might keep similar standing genetic variation. Alternatively, and not mutually exclusive, alleles underlying eye and pigment reduction may have pleiotropic effects that confer advantages in those ecological conditions that are common for all investigated populations, such as constant darkness or buffered seasonality (Pipan and Culver, 2012; Fišer et al., 2023). In particular, this could explain the repeated selection of variants (e.g., orange and red eye pigment, stellate head pigmentation pattern), that remain phenotypically masked in cave animals by the unpigmented phenotype.

Ultimately, a full understanding of repeated evolution in the *A. aquaticus* species complex will require identification of the exact genes and mutations underlying these traits. Such knowledge will clarify whether causative variation is still present in surface populations, reveal potential pleiotropic effects that drive repeated selection, and explain unusual genotypes observed in our crosses. Coupling this with forthcoming surface and cave genomes (Baković et al., 2021; Thomas et al., 2025) will enable tests of structural differences between cave and surface genomes and help resolve the role of genomic architecture in shaping eye and pigmentation phenotypes.

## Supplementary information

Supplementary figure
