# Supplementary material for: Genetic commonalities in eye and pigment evolution in the Asellus aquaticus species complex extend to the biogeographically and ecologically most divergent cave population, A. infernus
Source: Heredity (Edinb). 2026 Jul 22;135(9):740–9. doi: 10.1038/s41437-026-00868-z (PMC13612523; doi:10.1038/s41437-026-00868-z)
Supplement: Supplementary file 1 [file 41437_2026_868_MOESM1_ESM.pdf]

Supplementary figure:

```
SurfA      GGATAGCACTTATATCTCGAGAGATATATATTGTCGCGG---------------------
InfA       GGATAGCACTTTTATTTCGTGAAAGTTATAAATTGCCGCGTTTTATATTTTTAAAACCAG
SurfB      GGATAGCACT--TATTTCATGAAAGTTATAAATTGCCGCGTTTTATATTTTTCAAACCAG
InfB       GGATAGCACT--TATTTCGTGAAAGTTATAAATTGCCGCGTTTTATATTTTTCAAACCAG
           **********  *** **  ** *  ****   *  **
```

Figure S1. Alignment of the four *pax2* alleles present in the parents of the *red-eyeless* × *surface* F_2 *cross*. InfA and InfB were identified in the F_2 female parent (red eye pigmentation, no ommatidia), while SurfA and SurfB were identified in the surface male parent. Asterisks (*) denote identical nucleotides; dashes (–) indicate missing nucleotides.
